# 1-Methyl­sulfon­yl-4-nitro­benzene

**DOI:** 10.1107/S1600536808035861

**Published:** 2008-11-08

**Authors:** Dong-Sheng Ma

**Affiliations:** a College of Chemistry and Materials Science, Heilongjiang University, Harbin 150080, People’s Republic of China

## Abstract

In the title compound, C_7_H_7_NO_4_S, the nitro group is twisted by 10.2 (5) ° out of the plane of the benzene ring. Inversion-related mol­ecules are linked by non-classical C—H⋯O hydrogen bonds into dimers featuring an *R* ^2^_2_(10) motif.

## Related literature

For the synthesis, see: Nobles & Thompson (1965 ▶). For the supra­molecular patterns of nitro­phenyl compounds, see Glidewell *et al.* (2002 ▶); Ma (2007 ▶).
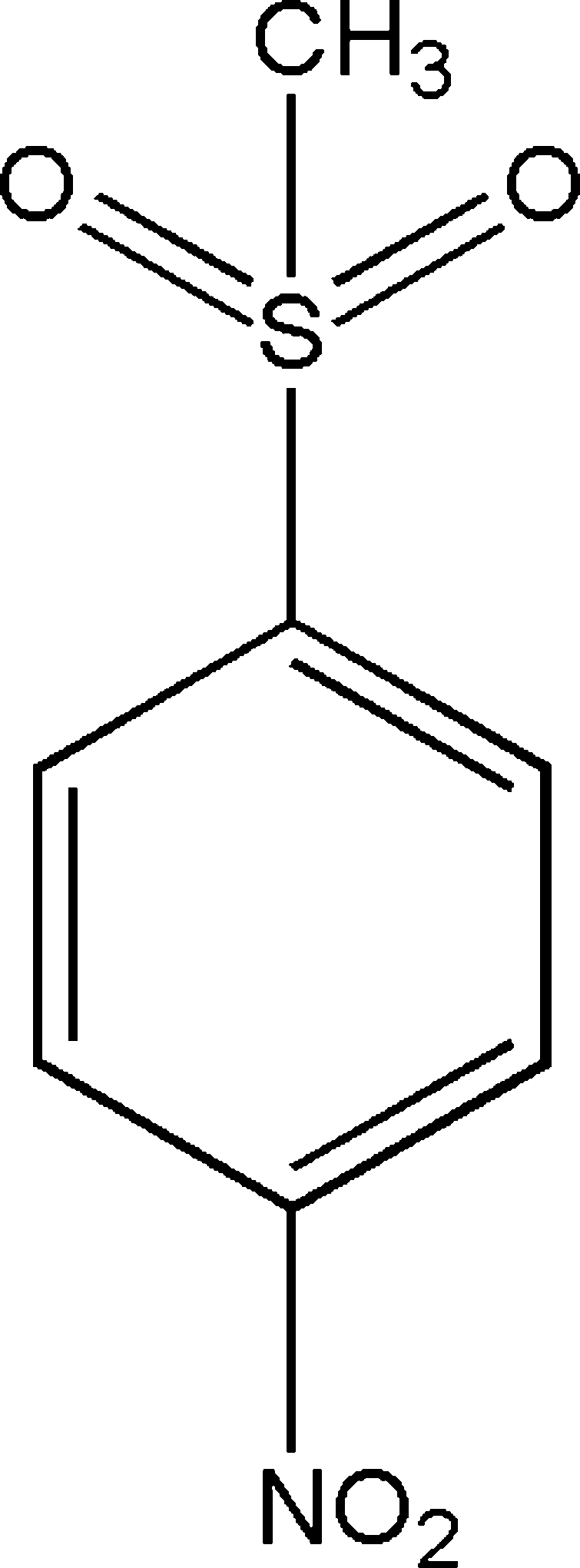


## Experimental

### 

#### Crystal data

C_7_H_7_NO_4_S
*M* *_r_* = 201.20
Monoclinic, 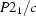

*a* = 6.3765 (13) Å
*b* = 8.0411 (16) Å
*c* = 16.426 (3) Å
β = 91.67 (3)°
*V* = 841.9 (3) Å^3^
*Z* = 4
Mo *K*α radiation
μ = 0.36 mm^−1^
*T* = 291 (2) K
0.21 × 0.19 × 0.16 mm

#### Data collection

Rigaku R-AXIS RAPID diffractometer
Absorption correction: multi-scan (*ABSCOR*; Higashi, 1995 ▶) *T*_min_ = 0.926, *T*_max_ = 0.942
7967 measured reflections
1933 independent reflections
1045 reflections with *I* > 2σ(*I*)
*R*_int_ = 0.042

#### Refinement

*R*[*F* ^2^ > 2σ(*F* ^2^)] = 0.042
*wR*(*F* ^2^) = 0.152
*S* = 1.11
1933 reflections
119 parameters
6 restraints
H-atom parameters constrained
Δρ_max_ = 0.32 e Å^−3^
Δρ_min_ = −0.45 e Å^−3^

### 

Data collection: *RAPID-AUTO* (Rigaku Corporation, 1998 ▶); cell refinement: *RAPID-AUTO*; data reduction: *CrystalStructure* (Rigaku/MSC, 2002 ▶); program(s) used to solve structure: *SHELXS97* (Sheldrick, 2008 ▶); program(s) used to refine structure: *SHELXL97* (Sheldrick, 2008 ▶); molecular graphics: *SHELXTL* (Sheldrick, 2008 ▶); software used to prepare material for publication: *SHELXL97*.

## Supplementary Material

Crystal structure: contains datablocks I. DOI: 10.1107/S1600536808035861/ng2509sup1.cif

Structure factors: contains datablocks I. DOI: 10.1107/S1600536808035861/ng2509Isup2.hkl

Additional supplementary materials:  crystallographic information; 3D view; checkCIF report

## Figures and Tables

**Table 1:** Hydrogen-bond geometry (Å, °)

*D*—H⋯*A*	*D*—H	H⋯*A*	*D*⋯*A*	*D*—H⋯*A*
C3—H2⋯O4^i^	0.93	2.65	3.462 (5)	147

Symmetry code: (i) 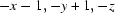.

**Fig. 1.**
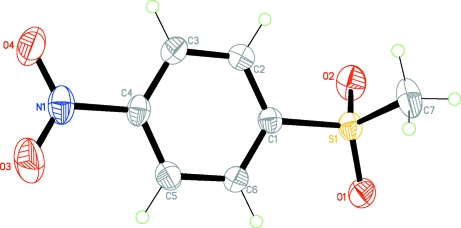
The molecular structure of (I), showing displacement ellipsoids at the 30% probability level for non-H atoms.

**Fig. 2.**
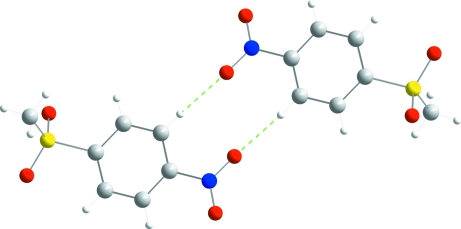
A view of the hydrogen-bonded (dashed lines) dimer.

## supplementary crystallographic information

### Comment

Simple carboxylic acids containing the nitrophenyl group exhibit a variety of supramolecular aggregation patterns (Glidewell *et al.*, 2002). We had reported the crystal structure of (2-nitrophenylsulfinyl)acetic acid in our previous work (Ma, 2007). In our attempt to synthesize the homologous compound of it, we unexpectedly obtain the title compound, (I), which is prepared by the decarboxylated reaction of (4-nitrophenylsulfonyl)acetic acid.

In (Fig. 1), all bond lengths and angles are normal. The nitro group is twisted out the phenylene ring by 10.2 (5) °.

A centrosymmetric dimer, containing an *R*^2^_2_(10) motif, is built up by C—H···O hydrogen bonding interactions between the phenyl and nitryl (Fig.2; table 1).

### Experimental

4-Nitrophenylthioacetic acid was prepared by nucleophilic reaction of chloroacetic acid (9.4 g, 0.1 mol) and 4-nitrothiophenol (15.5 g, 0.1 mol) under basic conditions. 4-Nitrophenylthioacetic acid (21.3 g, 0.1 mol) was then oxidized using 30% aqueous hydrogen peroxide (30 ml)in acetic anhydride solution (50 ml) (Nobles *et al.*, 1965). Unexpectedly product was obtained, namely 1-(methylsulfonyl)-4-nitrobenzene, which formed by the (4-nitrophenylsulfonyl)acetic acid decarboxylation under excessive hydrogen peroxide conditions.

### Refinement

All H atoms were placed in calculated positions and treated as riding on their parent atoms, with C—H = 0.93 Å (aromatic), *U*_iso_(H) = 1.2*U*_eq_(C); C—H = 0.96 Å (methyl), *U*_iso_(H) = 1.5*U*_eq_(C).

### Crystal data

**Table:**

C_7_H_7_NO_4_S	*F*_000_ = 416
*M**_r_* = 201.20	*D*_x_ = 1.587 Mg m^−^^3^
Monoclinic, *P*2_1_/*c*	Mo *K*α radiation λ = 0.71073 Å
Hall symbol: -P 2ybc	Cell parameters from 4509 reflections
*a* = 6.3765 (13) Å	θ = 3.2–27.5º
*b* = 8.0411 (16) Å	µ = 0.36 mm^−^^1^
*c* = 16.426 (3) Å	*T* = 291 (2) K
β = 91.67 (3)º	Block, yellow
*V* = 841.9 (3) Å^3^	0.21 × 0.19 × 0.16 mm
*Z* = 4	

### Data collection

**Table:**

Rigaku R-AXIS RAPID diffractometer	1933 independent reflections
Radiation source: fine-focus sealed tube	1045 reflections with *I* > 2σ(*I*)
Monochromator: graphite	*R*_int_ = 0.042
*T* = 291(2) K	θ_max_ = 27.5º
ω scan	θ_min_ = 3.2º
Absorption correction: Multi-scan(ABSCOR; Higashi, 1995)	*h* = −8→8
*T*_min_ = 0.926, *T*_max_ = 0.942	*k* = −10→9
7967 measured reflections	*l* = −21→20

### Refinement

**Table:**

Refinement on *F*^2^	Secondary atom site location: difference Fourier map
Least-squares matrix: full	Hydrogen site location: inferred from neighbouring sites
*R*[*F*^2^ > 2σ(*F*^2^)] = 0.042	H-atom parameters constrained
*wR*(*F*^2^) = 0.152	*w* = 1/[σ^2^(*F*_o_^2^) + (0.0576*P*)^2^ + 0.5194*P*] where *P* = (*F*_o_^2^ + 2*F*_c_^2^)/3
*S* = 1.12	(Δ/σ)_max_ < 0.001
1933 reflections	Δρ_max_ = 0.32 e Å^−^^3^
119 parameters	Δρ_min_ = −0.45 e Å^−^^3^
6 restraints	Extinction correction: none
Primary atom site location: structure-invariant direct methods	

### Special details

**Table:**

Geometry. All e.s.d.'s (except the e.s.d. in the dihedral angle between two l.s. planes) are estimated using the full covariance matrix. The cell e.s.d.'s are taken into account individually in the estimation of e.s.d.'s in distances, angles and torsion angles; correlations between e.s.d.'s in cell parameters are only used when they are defined by crystal symmetry. An approximate (isotropic) treatment of cell e.s.d.'s is used for estimating e.s.d.'s involving l.s. planes.
Refinement. Refinement of *F*^2^ against ALL reflections. The weighted *R*-factor *wR* and goodness of fit *S* are based on *F*^2^, conventional *R*-factors *R* are based on *F*, with *F* set to zero for negative *F*^2^. The threshold expression of *F*^2^ > σ(*F*^2^) is used only for calculating *R*-factors(gt) *etc*. and is not relevant to the choice of reflections for refinement. *R*-factors based on *F*^2^ are statistically about twice as large as those based on *F*, and *R*- factors based on ALL data will be even larger.

### Fractional atomic coordinates and isotropic or equivalent isotropic displacement parameters (Å^2^)

**Table:**

	*x*	*y*	*z*	*U*_iso_*/*U*_eq_	
C1	0.1240 (5)	0.8495 (4)	0.09033 (17)	0.0426 (7)	
C2	−0.0744 (5)	0.7940 (5)	0.1076 (2)	0.0546 (9)	
H1	−0.1268	0.8094	0.1593	0.066*	
C3	−0.1953 (6)	0.7154 (4)	0.0478 (2)	0.0548 (9)	
H2	−0.3297	0.6777	0.0584	0.066*	
C4	−0.1117 (5)	0.6945 (4)	−0.02732 (18)	0.0444 (8)	
C5	0.0841 (5)	0.7492 (4)	−0.04584 (19)	0.0523 (9)	
H3	0.1355	0.7340	−0.0977	0.063*	
C6	0.2042 (5)	0.8273 (4)	0.01402 (18)	0.0480 (8)	
H4	0.3384	0.8648	0.0030	0.058*	
C7	0.3546 (7)	0.7850 (5)	0.2321 (2)	0.0716 (12)	
H5	0.4392	0.8278	0.2767	0.107*	
H6	0.4342	0.7053	0.2024	0.107*	
H7	0.2320	0.7323	0.2528	0.107*	
N1	−0.2387 (6)	0.6099 (3)	−0.09136 (19)	0.0586 (8)	
O1	0.4601 (4)	1.0152 (3)	0.13042 (15)	0.0703 (8)	
O2	0.1478 (4)	1.0591 (3)	0.21070 (16)	0.0754 (8)	
O3	−0.1539 (5)	0.5713 (4)	−0.15365 (18)	0.0873 (10)	
O4	−0.4225 (5)	0.5820 (4)	−0.07856 (17)	0.0822 (9)	
S1	0.27883 (14)	0.94820 (10)	0.16746 (5)	0.0497 (3)	

### Atomic displacement parameters (Å^2^)

**Table:**

	*U*^11^	*U*^22^	*U*^33^	*U*^12^	*U*^13^	*U*^23^
C1	0.0461 (18)	0.0391 (16)	0.0423 (16)	0.0029 (13)	−0.0008 (13)	0.0050 (13)
C2	0.051 (2)	0.068 (2)	0.0453 (17)	−0.0034 (17)	0.0038 (15)	0.0008 (16)
C3	0.046 (2)	0.063 (2)	0.0553 (19)	−0.0115 (17)	−0.0019 (16)	0.0019 (17)
C4	0.0487 (19)	0.0360 (15)	0.0475 (17)	0.0011 (14)	−0.0129 (14)	0.0033 (14)
C5	0.055 (2)	0.061 (2)	0.0411 (16)	0.0026 (17)	−0.0005 (15)	−0.0022 (16)
C6	0.0415 (18)	0.0573 (19)	0.0452 (17)	−0.0024 (15)	−0.0016 (14)	0.0034 (15)
C7	0.096 (3)	0.065 (2)	0.053 (2)	−0.004 (2)	−0.025 (2)	0.0075 (18)
N1	0.074 (2)	0.0427 (15)	0.0574 (18)	−0.0008 (15)	−0.0218 (16)	0.0021 (14)
O1	0.0643 (17)	0.0840 (18)	0.0619 (14)	−0.0326 (14)	−0.0080 (12)	0.0045 (14)
O2	0.083 (2)	0.0676 (16)	0.0756 (17)	0.0135 (15)	−0.0062 (14)	−0.0285 (14)
O3	0.104 (2)	0.086 (2)	0.0707 (18)	−0.0009 (17)	−0.0156 (17)	−0.0348 (16)
O4	0.073 (2)	0.093 (2)	0.0790 (18)	−0.0289 (16)	−0.0244 (15)	0.0059 (16)
S1	0.0566 (6)	0.0457 (5)	0.0461 (5)	−0.0040 (4)	−0.0074 (4)	−0.0011 (4)

### Geometric parameters (Å, °)

**Table:**

C1—C2	1.378 (5)	C5—H3	0.9300
C1—C6	1.379 (4)	C6—H4	0.9300
C1—S1	1.771 (3)	C7—S1	1.747 (4)
C2—C3	1.384 (5)	C7—H5	0.9600
C2—H1	0.9300	C7—H6	0.9600
C3—C4	1.369 (5)	C7—H7	0.9600
C3—H2	0.9300	N1—O3	1.212 (4)
C4—C5	1.367 (5)	N1—O4	1.218 (4)
C4—N1	1.475 (4)	O1—S1	1.427 (3)
C5—C6	1.380 (4)	O2—S1	1.425 (3)
			
C2—C1—C6	120.8 (3)	C5—C6—H4	120.2
C2—C1—S1	119.6 (2)	S1—C7—H5	109.5
C6—C1—S1	119.6 (2)	S1—C7—H6	109.5
C1—C2—C3	119.8 (3)	H5—C7—H6	109.5
C1—C2—H1	120.1	S1—C7—H7	109.5
C3—C2—H1	120.1	H5—C7—H7	109.5
C4—C3—C2	118.2 (3)	H6—C7—H7	109.5
C4—C3—H2	120.9	O3—N1—O4	123.6 (3)
C2—C3—H2	120.9	O3—N1—C4	118.1 (3)
C5—C4—C3	122.9 (3)	O4—N1—C4	118.2 (3)
C5—C4—N1	118.4 (3)	O2—S1—O1	118.03 (18)
C3—C4—N1	118.6 (3)	O2—S1—C7	108.81 (19)
C4—C5—C6	118.6 (3)	O1—S1—C7	109.2 (2)
C4—C5—H3	120.7	O2—S1—C1	108.31 (16)
C6—C5—H3	120.7	O1—S1—C1	107.79 (15)
C1—C6—C5	119.6 (3)	C7—S1—C1	103.70 (16)
C1—C6—H4	120.2		

### Hydrogen-bond geometry (Å, °)

**Table:**

*D*—H···*A*	*D*—H	H···*A*	*D*···*A*	*D*—H···*A*
C3—H2···O4^i^	0.93	2.65	3.462 (5)	147

Symmetry codes: (i) −*x*−1, −*y*+1, −*z*.
